# Investigating Iranian English language teachers’ wellbeing across their career stages

**DOI:** 10.3389/fpsyg.2023.1275219

**Published:** 2024-01-08

**Authors:** Leila Ashegh Navaie, Javad Gholami, Zhila Mohammadnia

**Affiliations:** Department of English Language, Urmia University, Urmia, Iran

**Keywords:** career stages, experienced teachers, novice teachers, positive psychology, teacher wellbeing

## Abstract

This study aims to investigate the wellbeing of Iranian English as Foreign Language (EFL) teachers across their career stages in terms of teaching experience. In this regard, the study categorized teachers into three groups based on their experience: novice, experienced, and highly experienced. Using a quantitative and cross-sectional survey design, this study employed a validated scale to measure psychological wellbeing, positive perception about self and life, goal setting and time management, and positive relationships among teachers. Using a convenience sampling procedure, 182 teachers participated in the study, representing varying age ranges, teaching experience, and employment status. The data was collected through an online survey within a month and underwent descriptive and inferential statistical analyses using SPSS software. The results revealed significant differences in the wellbeing of language teachers across experience groups. More specifically, novice teachers scored lower in various wellbeing dimensions than experienced and highly experienced teachers. These findings emphasize the importance of considering career stages when investigating teachers’ professional wellbeing. The findings also contribute to a deeper understanding of the relationship between teaching experience and wellbeing, with implications for supporting the EFL teachers’ wellbeing and informing educational policies and practices. The study results are discussed, and the related implications are provided for running professional development courses to enhance teachers’ wellbeing.

## Introduction

In recent years, the increased teacher attrition and burnout rate have motivated many researchers to explore the range of factors that increase the possibility of these negative occurrences among teachers (Borman and Dowling, 2008; Weiland, 2021). Such professional happenings have partly been associated with the teachers’ overall wellbeing in that good feelings of teachers function as a strong factor in preventing teachers from leaving their jobs and from feeling less demotivated (Mercer, 2020; Chamani et al., 2023; Sulis et al., 2023). From this perspective, wellbeing has been conceptualized as a strong factor in the way teachers construct their identities, the range of emotions they experience, and the general membership teachers claim in their organizations (Talbot and Mercer, 2018; Hofstadler et al., 2021; Jin et al., 2021; Nazari and Xodabande, 2022).

Recent discussions of teacher wellbeing have called for more examination of the concept in relation to different factors shaping teachers’ overall sense of wellbeing. In this regard, Sulis et al. (2023) edited a volume that was structured around teachers’ career stages and the role of such stages in their wellbeing. They further called for further exploring this dimension of language teacher wellbeing. In response to this call for investigation, the present study explored the role of teaching experience in Iranian English as Foreign Language (EFL) teachers’ wellbeing. Teaching experience has been argued as a significant factor that shapes different dimensions of teachers’ professional growth (Tsui, 2009; Li, 2020). However, how teaching experience is associated with teachers’ wellbeing is lacking in the literature. Exploring this issue is significant because it provides implications for teacher educators to account for different teachers’ wellbeing across their stages of professional development and design teacher education courses that deal with such issues. Thus, the purpose of the present study is to explore the role of teaching experience in Iranian EFL teachers’ wellbeing by addressing the following question:

Are there any significant differences among novice, experienced, and highly experienced teachers in terms of their wellbeing?

## Literature review

### Language teacher wellbeing

More than two decades ago, Maslach and Leiter (1999) argued that “the most valuable and costly part of an education system are the people who teach. Maintaining their wellbeing and their contribution to student education should be a primary objective of educational leaders” (p. 303). The interest in teacher wellbeing has thus motivated many researchers to connect wellbeing to several professional challenges that shape teacher professionalism. For example, Mercer (2020) discusses how wellbeing influences teachers’ emotional labor and self-efficacy and that the absence of these factors complicates their job. Moreover, Sulis et al. (2023) discuss how social, cultural, political, and educational factors facilitate and/or impede teachers’ wellbeing and the range of emotional fluctuations they experience over their professional lives.

Wellbeing has mainly been studied from subjective and psychological or hedonic and eudemonic perspectives. Subjective wellbeing “is an umbrella term used to describe the level of wellbeing people experience according to their subjective evaluations of their lives” (Diener and Ryan, 2009, p. 391) and psychological wellbeing concerns seeking meaning in life and self-actualization (Gregersen et al., 2020; Greenier et al., 2021). These perspectives were first theorized by Ryan and Deci (2001) and have formed the major basis on which teachers’ professional characteristics as informing their wellbeing have been researched over the past decades. Indeed, the significance of wellbeing in teachers’ professional life has been argued as, “teachers who enjoy high levels of wellbeing are likely to be successful teachers, more engaged with their language teaching practice, and better able to face challenges that occur along the way” (p. 427).

In recent years, research has extensively explored language teachers’ wellbeing in different educational contexts (Mercer, 2020; Greenier et al., 2021; Hofstadler et al., 2021; Jin et al., 2021; Nazari and Xodabande, 2022). For example, Mercer (2020) explored eight Maltese teachers’ wellbeing in the country’s private sector based on an ecological perspective. Mercer collected data from different sources, including questionnaires, interviews, and prompts. Data analysis revealed that different ecologies of the context, including classroom, institutional, and social factors, played a significant role in the teachers’ wellbeing relative to those ecologies. Moreover, Nazari and Xodabande (2022) explored the wellbeing and professional identity construction of eight Iranian teachers through the lens of self-determination theory. The study findings showed that across the competence, autonomy, and relatedness components, the teachers faced tensions that complicated both feeling well and constructing their identities as effective professionals.

### Teaching experience

A central component in teaching and teacher education literature has been the role that individuals’ history and accrued experience play in their current functioning and future performances. For example, in his seminal paper on language teacher cognition, Borg (2003) identified experience as one of the major factors shaping teachers’ professional growth. In a somewhat theoretical account pointing to the role of reflection, Widdowson (2003) stated that “…experience itself teaches you nothing directly—you have to learn *from* it, indirectly—and this means discovering something beyond appearances, abstracting something general from particulars.” Similar observations have been made by Li (2020) discussing the significant role that experience plays in teachers’ personal development, ability to integrate different professional and institutional contextualities, and delivering quality instruction in a way conducive to student learning. Additionally, recent observational and experimental studies indicated that experience is closely related to perceived job satisfaction and self-efficacy among teachers, and more experienced teachers face fewer problems in classroom management and dealing with student misbehavior (Apter et al., 2020; Sulla and Rollo, 2023).

As a major conceptualizer of teaching experience, Tsui (2009) distinguished expert performance from expertise by highlighting that the former is “a *state* that is reached after years of experience and thousands of hours of practice,” while expertise involves “the *processes* which mediate or support experts’ superior performance” (p. 184). This discussion has, thus, led to theorizing similarities and differences between novice and experienced language teachers. For example, Tsui (2009) characterizes experienced teachers as those with a higher level of spontaneity in performance and a better ability to integrate different institutional factors. Moreover, Li (2020) argued that while novice teachers may be more into trying out novel ways of practicing, experienced teachers have a greater repertoire of instructional skills to guide their professional practices at the classroom and institutional levels. Hence, such similarities and differences have led to the burgeoning of research on different dimensions of novice and experienced teachers’ professionalism.

Motivated by this body of knowledge, research has explored novice and experienced teachers’ professionalism over the past decades (Burkhauser and Lesaux, 2017; Fallah and Nazari, 2019; Karimi and Norouzi, 2019; Nazari and Alizadeh Oghyanous, 2021). For example, Karimi and Norouzi (2019) divided 20 teachers into five groups based on different levels of their teaching experience and explored their pedagogical thought units. The study’s results showed “significant differences across th‑e five groups both in the sums of frequencies of pedagogical thought units produced by the teachers and in a number of the dominant pedagogical thought categories” (p. 1). Moreover, Nazari and Alizadeh Oghyanous (2021) explored the differences between novice and experienced teachers across their occupational stress/turnover intentions and grit/psychological wellbeing. Data were collected from 325 teachers, followed by interviews with 20 teachers. The study findings showed significant relationships between the four abovementioned constructs. Moreover, the qualitative findings showed that “while occupational stress collectively influences the teachers’ identity, emotions, and retaining in the profession, their wellbeing was largely defined in light of various institutional and socio-economic factors” (p. 1). The authors argued for more explorations into the role of experience in teachers’ professional characteristics, especially wellbeing, which is the purpose of this study.

### Purpose of the study

In their discussion of language teacher wellbeing, Sulis et al. (2023) called for more studies on the role of professional characteristics in teachers’ professional wellbeing. The present study explores this aspect of Iranian EFL teachers’ wellbeing in relation to their experience. Additionally, although research (e.g., Nazari and Alizadeh Oghyanous, 2021) has explored the role of experience in teacher wellbeing, it (1) needs more attention from the researchers to unpack more dimensions of this work and (2) that study has only divided teachers into two novice and experienced groups. The present study divides the teachers into three groups: novice, experienced, and highly experienced. This could better account for the career-stage perspective that Sulis et al. (2023) argued for in investigating teachers’ wellbeing. Thus, the present study explores Iranian EFL teachers’ wellbeing in relation to their stages of professionalism.

## Methods

### Research design

The study adopted a cross-sectional survey design to explore the wellbeing among Iranian EFL teachers at various career stages (Dörnyei, 2007). This design was chosen for its efficacy in capturing a snapshot of a phenomenon at a particular point in time, allowing for simultaneously analyzing data from a diverse range of participants (Ary et al., 2014). The survey instrument, as described below, was distributed online to accommodate the widespread geographical location of the participants and ensure a timely and cost-effective data collection process.

### Participants and context

In the context of Iran, English as a Foreign Language (EFL) teachers typically embark on their educational journey with a Bachelor’s degree in English Language Teaching, English Literature, or Translation Studies, often obtained from one of the numerous universities across the country that offer such programs. Many continue to refine their pedagogical expertise with Master’s degrees, and a significant number pursue Doctorates, emphasizing applied linguistics or TEFL (Teaching English as a Foreign Language). As for their work conditions, Iranian EFL teachers operate within diverse environments ranging from public schools, where large class sizes and limited resources challenge their instructional capabilities, to private language institutes and universities, where conditions and facilities are generally more conducive to language teaching and learning. Teachers often have smaller classes and access to more advanced teaching aids in these contexts.

The study participants were 182 Iranian EFL teachers recruited through convenience sampling based on their availability and willingness to participate. This non-probability sampling technique was utilized due to its practicality and efficiency in reaching a sizable number of respondents within the constraints of the study’s operational framework. The study’s inclusion criteria stipulated that participants must be currently active Iranian EFL teachers, possessing at least a Bachelor’s degree in a field relevant to English language education. Participants represented varying age ranges (*M* = 28, *SD* = 7), teaching experience, and employment status. More specifically, 42.3% (*N* = 77) of the participants were males, and 57.7% (*N* = 105) were females. Concerning the employment status, around 52% of the teachers were working only in private institutes (*N* = 95), 31% were employed by state-run schools (*N* = 57), and 16% were working both in private and state-run settings (*N* = 29). As for teaching experience, 33% were novice teachers having less than three years of teaching experience (*N* = 60), 30% were experienced teachers (3–10 years, *N* = 55), and 37% were highly experienced teachers (*N* = 67). In order to comply with ethical considerations in educational research, the participants provided their informed consent, and their anonymity was ensured in data collection.

### Instruments

The study employed a validated scale developed by Singh et al. (2016) to measure the participants’ (1) psychological wellbeing, (2) positive perception about self and life, (3) goal setting and time management, and (4) positive relationships. The first factor, consisting of nine items, pertains to an individual’s capacity to maintain equilibrium between their personal and professional lives. Illustrative statements within this factor include expressions such as “I actively pursue personal growth,” “I effectively utilize my abilities,” “I perceive substantial personal development over time,” and “I attach significance to my work.” The second factor, comprising seven items, examines an individual’s self-perception and overall life satisfaction, encompassing past experiences, present circumstances, and overall life situations. Example statements encompassed by this factor include “Overall, I am content when considering my life and personal circumstances,” “I have reconciled with my past,” and “I feel a general sense of contentment with various aspects of my life.” The third factor, encompassing eight items, assesses an individual’s aptitude for future planning and efficient time and task management. Representative items within this factor encompass expressions like “I derive satisfaction from setting goals and striving to accomplish them,” “I demonstrate competence in managing personal finances and affairs,” and “I exhibit adeptness in time management to ensure completion of necessary tasks.” The fourth factor, comprising five items, investigates the quality of an individual’s interpersonal relationships. Examples of items within this factor include statements such as “I experience mutually fulfilling relationships,” “I am widely perceived as affectionate and loving,” and “I possess a confidant with whom I can share intimate feelings.”

In the current study, the participants were administered the scale, where they indicated their level of agreement with each item using a Likert-type scale ranging from 1 (strongly disagree) to 5 (strongly agree). The comprehensive nature of the scale, encompassing the four factors, facilitates a multidimensional assessment of individuals’ wellbeing, enabling a comprehensive examination of diverse aspects contributing to their subjective wellbeing. Previous research has substantiated the reliability (Cronbach’s alpha = 0.93) and validity of the Wellbeing Scale, endorsing its appropriateness for evaluating wellbeing in diverse populations (Singh et al., 2016). The scale also showed an acceptable and high reliability index in this study (Cronbach’s alpha = 0.84). To ensure consistent and accurate data collection, participants were provided with explicit instructions regarding the purpose of the scale and the appropriate response format for each item.

### Data collection and analysis

Data collection was conducted over a period of 30 days to ensure ample response time for participants. The online survey was distributed using a locally popular social media network and posts on professional social media groups dedicated to Iranian EFL professionals. In doing so, invitations were sent to language teachers in social media platforms. The collected data was analyzed based on descriptive and inferential statistics. More specifically, the study focused on examining the differences in wellbeing among teachers with different levels of experience. Descriptive statistics were performed to obtain mean and standard deviations for participants’ scores using SPSS software version 25. The analysis of variance (ANOVA) was employed to compare the scores between the groups for all factors. Finally, multiple comparisons using the Bonferroni method were conducted to find specific pairwise differences between the groups.

## Results

The descriptive statistics for the participants’ wellbeing are represented in Table 1. The results revealed interesting patterns and differences across different groups. This study found that as teaching experience progressed from novice to experienced and highly experienced, there was a notable increase in overall psychological wellbeing. In this regard, novice teachers reported the lowest level of psychological wellbeing (*M* = 24.90, *SD* = 0.34), while experienced and highly experienced teachers had higher mean scores of 33.84 and 38.60, respectively. This suggests that with mounting years of teaching experience, teachers tend to experience greater psychological wellbeing. A similar trend was observed for positive perceptions about self and life. Novice teachers had the lowest mean score (*M* = 18.57, *SD* = 0.36) which increased to 22.93 for experienced teachers and 26.19 for highly experienced teachers. This indicates that as teachers gain more experience, they develop a more positive outlook toward themselves and their lives.

**Table 1:** Descriptive statistics.

Teaching experience	Psychological wellbeing	Positive perception about self and life	Goal setting and time management	Positive relationships	Overall wellbeing
Novice					
N	60	60	60	60	60
Minimum	20	14	16	6	64
Maximum	29	23	25	18	90
Mean	24.90	18.57	20.55	12.38	76.40
SD	0.345	0.368	0.346	0.429	0.717
Experienced					
N	55	55	55	55	55
Minimum	29	19	23	11	88
Maximum	38	28	32	23	110
Mean	33.84	22.93	27.78	17.25	101.80
SD	0.393	0.372	0.381	0.449	0.634
Highly experienced					
N	67	67	67	67	67
Minimum	34	22	18	9	89
Maximum	43	31	27	27	121
Mean	38.60	26.19	23.06	16.21	104.06
SD	0.318	0.384	0.312	0.551	0.879

In terms of *goal setting* and *time management*, experienced teachers demonstrated higher mean scores (27.78) compared to both novice (20.55) and highly experienced (23.06) teachers. This suggests that experience in the teaching profession may contribute to improved goal-setting abilities and effective time-management skills. The analysis of positive relationships among teachers also revealed interesting findings. Experienced teachers had a higher mean score (17.25) than both novice (12.38) and highly experienced (16.21) teachers. This implies that as teachers gain more experience, they tend to develop better positive relationships with others. However, it should be noted that the difference between experienced and highly experienced teachers in this aspect was minimal. Finally, an examination of the overall wellbeing showed that experienced (101.80) and highly experienced (104.06) teachers had higher mean scores compared to their novice colleagues (76.40). This suggests that as teachers accumulate more experience in their careers, they generally experience higher overall wellbeing. However, it is worth mentioning that the difference between experienced and highly experienced teachers in terms of overall wellbeing was not substantial.

**Figure 1:**
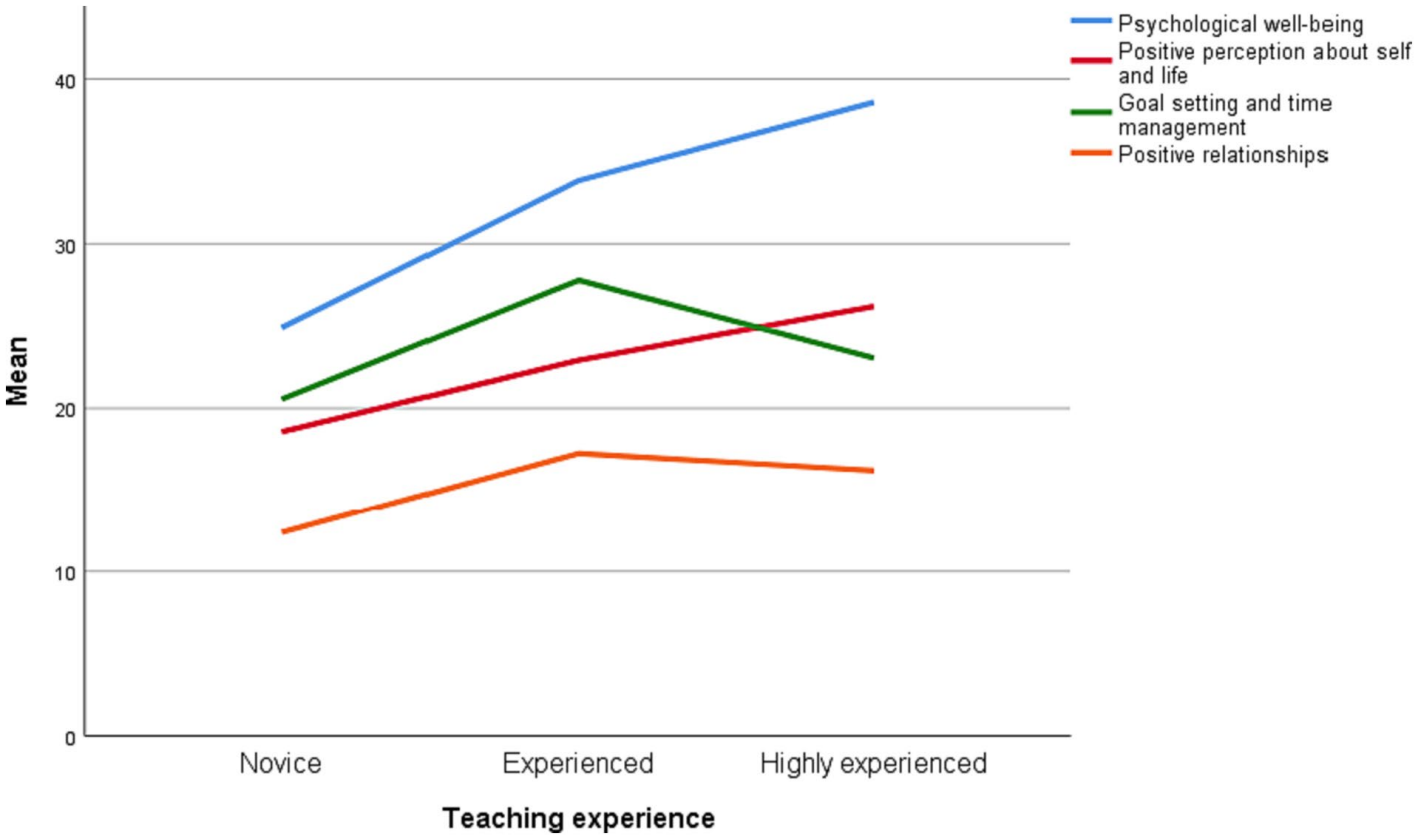
Wellbeing profiles of the novice, experienced, and highly experienced language teachers.

Figure 1 provides a visual representation of the wellbeing profiles of the novice, experienced, and highly experience language teachers. As shown below, there is a growing trend for language overall wellbeing as teachers gain more experience. However, in terms of *goal setting and time management*, and *positive relationships*, the experienced teachers had higher scores than novices and highly experienced teachers.

The analysis of variance (ANOVA) results revealed significant differences in factors related to wellbeing among teachers with different levels of teaching experience (Table 2). This analysis showed that teaching experience significantly impacted *psychological wellbeing*, *positive perception about self and life*, *goal setting and time management*, *positive relationships*, and *overall wellbeing* (*p* ≤ 0.001).

**Table 2:** ANOVA results for comparing groups.

	Sum of squares	df	Mean square	*F*	Sig.
Psychological wellbeing	6050.739	2	3025.370	407.466	0.000
Positive perception…	1845.849	2	922.925	107.211	0.000
Goal setting and…	1538.886	2	769.443	107.267	0.000
Positive relationships	775.646	2	387.823	26.786	0.000
Overall wellbeing	28699.901	2	14349.951	399.170	0.000

Table 3 summarizes the results from multiple comparisons using the Bonferroni method to examine the significant differences between the groups with varying experiences regarding various wellbeing-related factors. Regarding psychological wellbeing, the results indicate that novice teachers scored significantly lower than experienced and highly experienced teachers. Novice teachers had a mean difference of −8.936 compared to experienced teachers and −13.697 compared to highly experienced teachers. On the other hand, experienced teachers exhibited significantly higher psychological wellbeing scores than novices, with a mean difference of 8.936. However, their scores were lower than those of highly experienced teachers, showing a mean difference of −4.761. Highly experienced teachers demonstrated the highest psychological wellbeing scores, surpassing both novice and experienced teachers, with a mean difference of 13.697 and 4.761, respectively.

**Table 3:** Multiple comparisons for dependent variables based on teaching experience (Bonferroni).

Dependent variable	Teaching experience	Mean difference	SE	Sig.	95% confidence interval
Psychological wellbeing	Novice vs. experienced	−8.936*	0.509	0.000	−10.17 to −7.71
	Novice vs. highly experienced	−13.697*	0.484	0.000	−14.87 to −12.53
	Experienced vs. highly experienced	4.761*	0.496	0.000	3.56 to 5.96
Positive perception about self and life	Novice vs. Experienced	−4.361*	0.548	0.000	−5.68 to −3.04
	Novice vs. highly experienced	−7.627*	0.521	0.000	−8.89 to −6.37
	Experienced vs. highly experienced	3.267*	0.534	0.000	1.98 to 4.56
Goal setting and time management	Novice vs. experienced	−7.232*	0.500	0.000	−8.44 to −6.02
	Novice vs. highly experienced	−2.510*	0.476	0.000	−3.66 to −1.36
	Experienced vs. highly experienced	4.722*	0.487	0.000	3.54 to 5.90
Positive relationships	Novice vs. experienced	−4.871*	0.710	0.000	−6.59 to −3.15
	Novice vs. highly experienced	−3.826*	0.676	0.000	−5.46 to −2.19
	Experienced vs. highly experienced	1.046	0.692	0.398	−0.63 to 2.72
Overall wellbeing	Novice vs. experienced	−25.400*	1.119	0.000	−28.10 to −22.70
	Novice vs. highly experienced	−27.660*	1.066	0.000	−30.24 to −25.08
	Experienced vs. highly experienced	−2.260	1.091	0.119	−4.90 to 0.38

*The mean difference is significant at the 0.05 level.

Regarding positive perception about self and life, novice teachers reported significantly lower scores compared to both experienced and highly experienced teachers. The mean difference was −4.361 between novices and experienced teachers and −7.627 between novices and highly experienced teachers. Experienced teachers scored significantly higher than their novice counterparts, with a mean difference of 4.361 but lower than highly experienced ones, showing a mean difference of −3.267. Highly experienced teachers had the highest positive perception of self and life scores, exceeding novice and experienced ones, with a mean difference of 7.627 and 3.267, respectively.

In terms of goal setting and time management, novice teachers scored significantly lower than both experienced and highly experienced teachers. Novice teachers exhibited a mean difference of −7.232 compared to experienced teachers and −2.510 compared to highly experienced teachers. Experienced teachers had significantly higher scores than novices, with a mean difference of 7.232. However, their scores did not significantly differ from those of highly experienced teachers, with a mean difference of 4.722. Highly experienced teachers had higher scores in goal setting and time management compared to novices, with a mean difference of 2.510, but their scores did not significantly differ from those of experienced teachers.

Concerning positive relationships, novice teachers reported significantly lower scores compared to both experienced and highly experienced teachers. Novices had a mean difference of −4.871 compared to experienced teachers and −3.826 compared to highly experienced teachers. Experienced teachers scored significantly higher than novices, with a mean difference of 4.871. However, their scores did not significantly differ from those of highly experienced teachers. Highly experienced teachers had higher scores in positive relationships compared to novices, with a mean difference of 3.826. Nevertheless, their scores did not significantly differ from those of experienced teachers.

Finally, regarding overall wellbeing, novice teachers had significantly lower scores compared to experienced and highly experienced teachers. Novices had a mean difference of −25.400 compared to experienced teachers and −27.660 compared to highly experienced teachers. Experienced teachers scored significantly higher than novices, with a mean difference of 25.400. However, their scores did not significantly differ from those of highly experienced teachers. Highly experienced teachers had higher overall wellbeing scores compared to novices, with a mean difference of 27.660. Their scores also did not significantly differ from those of experienced teachers.

## Discussion

The present study explored the wellbeing of Iranian novice, experienced, and highly experienced teachers. The study’s results revealed that across all of the components of the teacher wellbeing scale, experienced teachers reported higher levels of wellbeing than novice ones. This finding aligns with the previous studies showing the higher levels of professional performance and cognitive functioning by experienced teachers compared to novice teachers. For example, Wolff et al. (2016) found that while novice teachers were more oriented toward behavioral issues in classroom management, experienced teachers were more concerned with the quality of learning in classroom management. This finding also aligns with those reported in Karimi and Norouzi (2019) regarding the experienced teachers’ higher levels of pedagogical thought units as compared to novice teachers.

This finding could be interpreted in light of the characteristics that Tsui (2009) discussed. She argued that experienced teachers are better able to “recognize patterns in classroom events very quickly, and they are able to interpret these patterns in meaningful ways because of the hundreds of hours that they have spent in the classroom” (pp. 192–193). From this perspective, it seems that experienced and highly experienced teachers, despite their trivial differences, are better able to draw insights from their professional work to contribute to their wellbeing. A similar observation has been made by Talbot and Mercer (2018) and Mercer (2020) regarding how teachers can draw on their own and surrounding potentials to facilitate the way for optimal levels of experiencing wellbeing. In this sense, it seems that experienced teachers can find more meaning in their classroom, institutional, and overall career growth in relation to their contributions to teachers’ wellbeing, which justifies why these teachers displayed higher levels of wellbeing compared to novice teachers.

Such a higher level of wellbeing among the teachers could also be observed in relation to the specific components of the scale. That is, experienced and highly experienced teachers outperformed the novices across the psychological wellbeing, positive perception about self and life, goal setting and time management, and positive relationships. All of these components match the characteristics that have been enumerated for experienced teachers, including further autonomy, more flexibility, better efficiency in lesson planning, and greater integrated knowledge (see Tsui, 2009; Li, 2020). That is, due to their increased knowledge about different educational issues, experienced teachers seem to be better able to situate such issues into the overall landscape of their professionalism, one which was related to their wellbeing in this study.

The findings of the study could be well interpreted in light of the career stage that Sulis et al. (2023) discussed regarding its meaning for teachers’ wellbeing. They argue that the stage that teachers are at bring to them a set of meaning-making processes that can significantly shape their wellbeing. For example, novice teachers can enjoy more concerns with students’ learning or experienced teachers can enjoy higher levels of wellbeing because they see themselves at higher performance levels. The current study’s findings add to this discussion by showing how novice, experienced, and highly experienced teachers differ in their wellbeing in relation to the stage of their carer. Thus, the study moves the discussion from theoretical to practical and documented levels by showing how career-stage differences shape teachers’ wellbeing.

However, there were differences between experienced and highly experienced teachers, with experienced teachers outperforming the highly experienced teachers across some levels of their wellbeing. There have been arguments that experience can ensure teachers’ effectiveness and better performance to a certain extent, and it is not that greater experience necessarily equals permanent better growth (Tsui, 2009; Harris and Sass, 2011). This finding is quite novel in the context of teacher wellbeing because it shows that even experienced teachers differ in their levels of wellbeing. This means that experience can ensure teacher wellbeing to a certain degree across teachers’ career growth. After some time, they are likely to be influenced by some internal and external factors negatively shaping their wellbeing. Thus, experience can play a key role in the differences between novice and experienced teachers and in experienced teachers’ wellbeing when interpreted within the groups.

### Implications

The outcomes of this investigation into the wellbeing of Iranian EFL teachers at varying career stages have significant implications for the design and delivery of professional development (PD) courses. Central to these implications is the recognition that teacher wellbeing is an essential factor influencing teachers’ daily experiences and professional performance (Mercer, 2020). As such, PD providers are prompted to consider wellbeing as a foundational component of their course design.

The importance of tailoring PD to career stages cannot be overstated. Novice teachers, who are often navigating the complexities of classroom management and curriculum delivery for the first time (Farrell, 2015), may require PD that supports resilience and stress management to cope with the initial challenges of the profession. Additionally, novice teachers could benefit from the strategies that foster self-efficacy and the ability to form supportive relationships with colleagues and students, thereby enhancing their overall wellbeing. For experienced teachers, PD might focus on sustaining motivation and passion for teaching, preventing burnout, and promoting career advancement. It is essential for PD courses for experienced teachers to provide a platform for sharing best practices, encouraging reflective teaching, and exploring advanced pedagogical strategies. This focus not only aids in maintaining their wellbeing but also in invigorating their instructional methods. For highly experienced teachers, PD courses could be geared toward leadership skills, mentoring new teachers, and contributing to curriculum development. Such roles can provide a renewed sense of purpose and fulfillment, positively impacting their wellbeing. These teachers, with their wealth of experience, can also be instrumental in shaping the institutional culture to prioritize and enhance the wellbeing of the teaching community.

Courses addressing wellbeing should integrate components that tackle in-class dynamics, institutional policies, and broader sociopolitical issues that impact teachers’ work lives. For instance, PD courses could include sessions on classroom management strategies that promote a positive learning environment, reducing stress and enhancing job satisfaction. Similarly, training that informs teachers about their rights, institutional support systems, and ways to navigate educational policies contributes to a sense of empowerment and wellbeing. Moreover, it is essential to recognize the socio-political context in which Iranian EFL teachers operate. Sociopolitical awareness and advocacy training can equip teachers to navigate better and influence the policies that frame their professional lives. Understanding the broader context in which they teach can also foster a sense of community and collective efficacy, a significant component of teacher wellbeing. In addition to content, the methodology of delivering these PD courses warrants careful consideration. Adult learning principles suggest that PD should be interactive, engaging, and directly relevant to teachers’ contexts. Facilitators should employ collaborative learning strategies, such as workshops, peer observations, and discussion forums, which can foster a supportive community of practice. Such methodologies aid in the immediate application of learned strategies and contribute to long-term professional growth and wellbeing. Lastly, the successful implementation of PD courses requires institutional backing. Schools and educational authorities should recognize the value of teacher wellbeing and support PD initiatives through funding, time allowances, and by creating a culture that encourages continuous professional growth.

## Conclusion

The findings of this study showed that experienced Iranian teachers enjoy higher levels of wellbeing compared to novice teachers and that experienced teachers also experience higher levels of wellbeing compared to highly experienced teachers. These findings extend the literature on teacher wellbeing by showing that experience plays a key role in Iranian teachers’ wellbeing across different career stages. In this regard, the findings provide original contributions on how a factor that lies beneath teachers’ daily and immediate professional work comes to significantly shape their professional growth.

The findings of the study provide implications for teacher educators to run professional development courses for teachers. In this regard, as wellbeing is an important factor in the way teachers experience wellbeing, teacher educators can run courses that cover the issues related to wellbeing including in-class, institutional, and sociopolitical factors. Such courses could be effectively run if they are tailored to teachers’ career stages. That is, a course that involves such issues should be anchored into novice and experienced teachers’ needs for wellbeing and the related factors. These courses are more likely to account for teachers’ wellbeing based on what they need at their current educational level. The benefit of such courses is that novice and experienced teachers can find the courses more relevant to their daily professional performances and how the course informs their practices.

The current study had limitations that should be addressed in future research. First, the study was conducted using only a questionnaire and it was cross-sectional. Future studies are suggested to employ more qualitative approaches to explore how teachers view their wellbeing relative to their respective career stage and capture nuances in their wellbeing change and development over time. Second, wellbeing could be directly related to teachers’ in-class performances. Thus, future researchers can use observational schemes to see how classroom events shape their interpersonal relationships and sense of wellbeing. However, it is hoped that teachers, both novice and experienced teachers, can benefit from the study findings to appreciate this issue further and find ways to boost their wellbeing.

## Data availability statement

The original contributions presented in the study are included in the article/supplementary material, further inquiries can be directed to the corresponding author.

## Ethics statement

Ethical approval was not required for the studies involving humans because in the context of the non-medical nature of this article, which does not involve sensitive human data, getting ethical approval was regarded unnecessary. The studies were conducted in accordance with the local legislation and institutional requirements. The participants provided their written informed consent to participate in this study.

## Author contributions

LA: Conceptualization, Data curation, Formal analysis, Investigation, Methodology, Writing – original draft. JG: Supervision, Investigation, Writing – original draft, Writing – review & editing. ZM: Reconceptualization, Co-supervision, Methodology, Resources, Writing – second draft, Writing - review & editing.
